# Analysis of the epidemiological characteristics of pulmonary tuberculosis in Shijiazhuang, China 2010–2023

**DOI:** 10.3389/fpubh.2025.1621695

**Published:** 2025-07-03

**Authors:** Xin Wang, Jianhua Guo, Xiaojing Shi, Lihua Cui

**Affiliations:** ^1^School of Public Health, North China University of Science and Technology, Tangshan, China; ^2^Department of Tuberculosis Control and Prevention, Shijiazhuang Center for Disease Control and Prevention, Shijiazhuang, China; ^3^Hebei Key Laboratory of Intractable Pathogens, Shijiazhuang, China

**Keywords:** pulmonary tuberculosis, Joinpoint regression model, epidemiological characteristics, spatial autocorrelation analysis, space-time scan analysis

## Abstract

**Background:**

Spatio-temporal analysis is a key epidemiological tool for monitoring disease transmission and identifying outbreak hotspots. However, the patterns of pulmonary tuberculosis (PTB) spread over time and space in Shijiazhuang remain poorly understood. This study aims to clarify the spatio-temporal dynamics of PTB transmission in this region.

**Methods:**

We conducted a retrospective study using PTB surveillance data from 2010 to 2023, extracted from the national Tuberculosis Information Management System. Descriptive epidemiological analysis was conducted to assess the severity and distribution characteristics of PTB in Shijiazhuang. The Joinpoint regression model was employed to analyze the annual temporal trends. Spatial autocorrelation analysis and Space–time scan analysis were utilized to explore the spatio-temporal clustering characteristics.

**Results:**

From 2010 to 2023, a total of 54,855 PTB cases were reported, with an average annual incidence of 38.97 per 100,000 population. Males, older adults, and farmers were disproportionately affected. The overall incidence declined significantly (AAPC = −7.65%, *p* < 0.05), with a steeper drop between 2010 to 2013 and a more gradual decline thereafter. Spatial analysis revealed persistent high-high clusters in rural counties such as Lingshou county and Pingshan county, and low-low clusters in central urban districts. The phased space–time scan analysis results identified 19 clusters.

**Conclusion:**

This study reveals a declining PTB incidence in Shijiazhuang, with a higher burden among males, older adults, and farmers, alongside persistent spatial clusters in rural areas, particularly in the north. These findings emphasize the need for targeted interventions and strengthened rural surveillance to achieve tuberculosis elimination goals.

## Introduction

Tuberculosis (TB) is a chronic infectious disease caused by *Mycobacterium tuberculosis* (MTB), which is mainly transmitted through the respiratory tract (1). When an infected person coughs, sneezes or talks, the droplets containing MTB will enter the air, and then be inhaled by others, causing infection. TB may invade various organs throughout the human body, but mainly affects the lungs, known as pulmonary tuberculosis (PTB) (2). Although TB can be prevented and treated, it is still a major global public health problem (3, 4). The latest Global Tuberculosis Report estimated 10.8 million new cases worldwide, with an incidence of 134 per 100,000—marking a continued rise since 2021 and suggesting tuberculosis may once again become the leading cause of death from a single infectious agent (5). In 2023, China ranked third among the 30 countries with the highest TB burden, with the TB prevention and control situation remaining serious (5).

Projections from research suggest that attaining the global TB targets by 2035 may be difficult for China under its current framework of prevention and control, thereby emphasizing the critical need for the development of novel strategies and methodologies (6). Geographic information systems and spatio-temporal clustering analysis are widely used in public health, particularly in the study of infectious diseases (7, 8). These methods help identify disease clusters, dynamically visualize changes in disease occurrence and cluster trends over time and space, and elucidate geographic distribution patterns and risk levels in different regions (9). Studies have shown that the incidence of PTB has spatial clustering and regional attributes (10, 11). A systematic review on the spatial clustering of TB in the global general population revealed that the high-risk attribute of TB is related to the spatial distribution of hotspots (12). Determining the geographical distribution of disease hotspots is helpful to identify areas with high incidence rate and formulate prevention and control measures (13).

As the capital of Hebei Province, China, Shijiazhuang is an important economic, cultural, and transportation hub in the northern China, characterized by high population density and significant mobility, which increases the risk of infectious disease transmission (14). However, the PTB situation in Shijiazhuang was not optimistic, with its incidence rate ranking first in the Beijing-Tianjin-Hebei region in 2023 (15, 16). Therefore, based on descriptive epidemiology, this study employs recent techniques such as temporal, spatial, and spatio-temporal analyses to precisely identify high-risk populations, times, and regions, thereby providing a scientific basis for the formulation of targeted prevention and control measures and contributing to the achievement of the 2035 tuberculosis control target.

## Materials and methods

### Study area

Shijiazhuang is located in the North China region and the central southern part of Hebei Province, between 37°27′~38°47′ north latitude and 113°30′~115°20′ east longitude. As the provincial capital city closest to the national capital Beijing, Shijiazhuang is not only a regional central city of the Beijing-Tianjin-Hebei world-class city cluster but also a national logistics hub. The total area is 15,800 square kilometers. The permanent population of Shijiazhuang is 11.23 million in 2023. Shijiazhuang has a temperate monsoon climate with four distinct seasons, an average annual temperature of 14.8°C, and an average annual precipitation of 741.4 millimeters (17). This study encompasses all 22 administrative divisions under Shijiazhuang’s jurisdiction, including districts, counties, and county-level cities.

### Data sources

PTB case data for Shijiazhuang from 2010 to 2023 were sourced from the Tuberculosis Information Management System (TBIMS) within the China Information System for Disease Control and Prevention (CISDP). This system monitors and covers all PTB case reports from hospitals and public health institutions across Chinese provinces and municipalities. Reported cases of PTB include laboratory confirmed and clinically diagnosed cases. The diagnostic criteria for tuberculosis were based on the National Health Commission of the People’s Republic of China WS 288-2008, WS 288-2017, and the Technical Guidelines for PTB Prevention and Control in China (18, 19). The population data of Shijiazhuang from 2010 to 2023 is sourced from the Shijiazhuang Municipal Bureau of Statistics. Vector maps are derived from National Catalogue Service for Geographic Information.

### Descriptive analysis

Descriptive epidemiology was applied to examine the characteristics of pulmonary tuberculosis (PTB) in Shijiazhuang from 2010 to 2023. Temporal trends were assessed using Joinpoint regression to estimate the annual percentage change (APC), average annual percentage change (AAPC), and their 95% confidence intervals (CI). If the APC/AAPC values are positive, it indicates an increasing trend; conversely, if they are negative, it indicates a decreasing trend (6). Seasonal variation was evaluated using a seasonal index, with values above 100% indicating months of heightened TB reporting (20).

### Spatial autocorrelation analysis

The spatial autocorrelation analysis is used to study whether an attribute value in the area has spatial correlation and correlation strength, including global spatial autocorrelation and local spatial autocorrelation. The commonly used index for global spatial autocorrelation is Moran’s *I*, which ranges from −1 to 1. A value of *I* > 0 indicates positive spatial autocorrelation (clumped distribution), with larger values signifying stronger spatial correlation; *I* < 0 indicates negative spatial autocorrelation (dispersed distribution), with smaller values indicating greater spatial differences; *I* = 0 denotes no spatial correlation (random distribution) (11). The formula for calculating Moran’s *I* is as follows:


$$I=\frac{n\sum_{i=1}^{n}\sum_{j=1}^{n}W_{\mathrm{ij}}(x_{i}-\bar{x})(x_{j}-\bar{x})}{\sum_{i=1}^{n}\sum_{j=1}^{n}W_{\mathrm{ij}}\sum_{i=1}^{n}{\left(x_{i}-\bar{x}\right)}^{2}}$$

Where *n* was the number of counties, *x_i_* and *x_j_* were the indicators of autocorrelations from unit index *i* and *j*, $\bar{x}$ was average and *w_ij_* was the matrix of spatial weights.

Local spatial autocorrelation analysis is employed to detect the spatial aggregation patterns between each region and its adjacent regions. It is presented through a clustering map of local indicators of spatial association (LISA), which encompasses four types: high-high value aggregation regions, high-low value aggregation regions, low-high value aggregation regions, and low-low value aggregation regions (18). The calculation method for the local Moran I is detailed as follows:


$$I_{i}=\frac{n(x_{i}-\bar{x})}{\sum_{j=1}^{n}{\left(x_{i}-\bar{x}\right)}^{2}}\sum_{j=1}^{n}w_{\mathrm{ij}}(x_{j}-x_{i})$$

In the above formulas, *n*, *x_i_*, *x_j_*, *w_ij_*, and $\bar{x}$ are the same as in the former formula.

### Space–time scan analysis

A Poisson distribution model is adopted for space–time scan analysis. The principle is to establish a cylindrical scanning window with the study area as the base and time as the height. As the window continuously changes, once it reaches the set upper limit value, the above steps are repeated. Based on the actual number of incidence cases and the theoretical number of incidence cases inside and outside the scanning window, the log-likelihood ratio (LLR) is calculated to evaluate the aggregation areas and aggregation time. The area with the maximum LLR value is classified as a primary aggregation area, and the rest are all secondary aggregation areas (21). In order to more accurately describe the spatial aggregation distribution characteristics of the reported PTB rate, this study divides the space–time analysis time into two stages: 2010–2017 and 2018–2023, in accordance with the revised diagnostic criteria for PTB. Regarding the choice of scanning window size, previous studies have proposed various approaches, primarily aiming to reduce overlapping clusters or to ensure that any single cluster does not exceed 15% of the total study area when applying irregular spatial scan statistics (22). In our analysis, we employed a purely spatial scan approach, systematically testing window sizes ranging from 5 to 50% of the total at-risk population, with 5% increments. Based on these comparisons, we selected a window size of 10% of the total population as optimal. The number of Monte Carlo replications was set at 999 to ensure robust statistical inference.

The formula for LLR is as follows:


$$\mathrm{LLR}=\frac{{\left(\frac{n_{z}}{u_{z}}\right)}^{n_{z}}{\left(\frac{n_{g}-n_{z}}{u_{g}-u_{z}}\right)}^{n_{g}-n_{z}}}{{\left(\frac{n_{g}}{u_{g}}\right)}^{n_{g}}}$$

Where 𝑛_𝑧_ represents the actual number of cases in the space-temporal window *Z*; 𝑢_𝑧_ represents the expected number of cases in the space-temporal window *Z* under the random assumption; 𝑛_𝑔_ represents the number of cases in the entire study area; and 𝑢_𝑔_ represents the total expected number of cases in the entire study area.

### Statistical software

Trend analysis of PTB was performed using the Joinpoint software (version 5.3.0; National Cancer Institute, Calverton, MD, United States). Geographic information software ArcGIS (version 10.1; Environmental Systems Research Institute, Inc., Redlands, CA, USA) was used for global and local spatial autocorrelation analysis. To identify spatio-temporal clusters of PTB, space–time scan statistics were applied using SaTScan software (version 10.1; Martin Kulldorff, Harvard Medical School). The scanning analysis was used to detect high-risk clusters by exploring both temporal windows and geographic regions. All statistical tests were two-sided, and a significance level of α = 0.05 was adopted.

## Results

### Epidemiological characteristics

Between 2010 and 2023, a total of 54,855 cases of PTB were reported in Shijiazhuang City, with an average annual incidence rate of 38.97 per 100,000 population. The annual incidence rates ranged from 22.10 per 100,000 to 66.37 per 100,000. Although the overall trend showed a decline of 40.75% in the annual reported incidence rate, there was a rebound in 2023 (Supplementary Figure S1) with a pronounced gender disparity. Among these, 36,865 were males and 17,990 were females, yielding a male-to-female ratio of 2.05:1. The average age of PTB patients was 45.18 years (0–102 years). Notably, individuals aged 65 years and older accounted for 20.76% of all reported cases, indicating a substantial burden of disease among the older adult. Regarding occupational distribution, the three most commonly affected groups were farmers (66.97%), students (9.05%), and individuals engaged in housework or unemployed (5.51%) (Table 1).

**Table 1:** Demographic characteristics analysis of pulmonary tuberculosis cases in Shijiazhuang from 2010 to 2023.

Variable	Case number	Percentage (%)
Gender		
Female	36,865	67.20
Male	17,990	32.80
Age		
0–15 year	965	1.76
16–20 year	5,332	9.72
21–25 year	6,651	12.12
26–30 year	5,167	9.42
31–35 year	3,651	6.66
36–40 year	2,811	5.12
41–45 year	2,966	5.41
46–50 year	3,749	6.83
51–55 year	4,065	7.41
56–60 year	4,742	8.64
61–65 year	4,319	7.87
66–70 year	3,700	6.75
71–75 year	2,912	5.31
≥76 year	3,825	6.97
Occupation		
Peasant	36,738	66.97
Student	4,962	9.05
Housework and unemployment	3,021	5.51
Retiree	2,640	4.81
Others	2,522	4.60
Worker	1,665	3.04
Cadres and staff members	1,057	1.93
Unknown	542	0.99
Commercial service	528	0.96
Medical staff	391	0.71
Teacher	317	0.58
Migrant worker	223	0.41
Food and beverage industry	119	0.22
Pastoralist	48	0.09
Scattered children	47	0.09
Seafarers and long-distance drivers	18	0.03
Public place service staff	17	0.03

**Figure 1:**
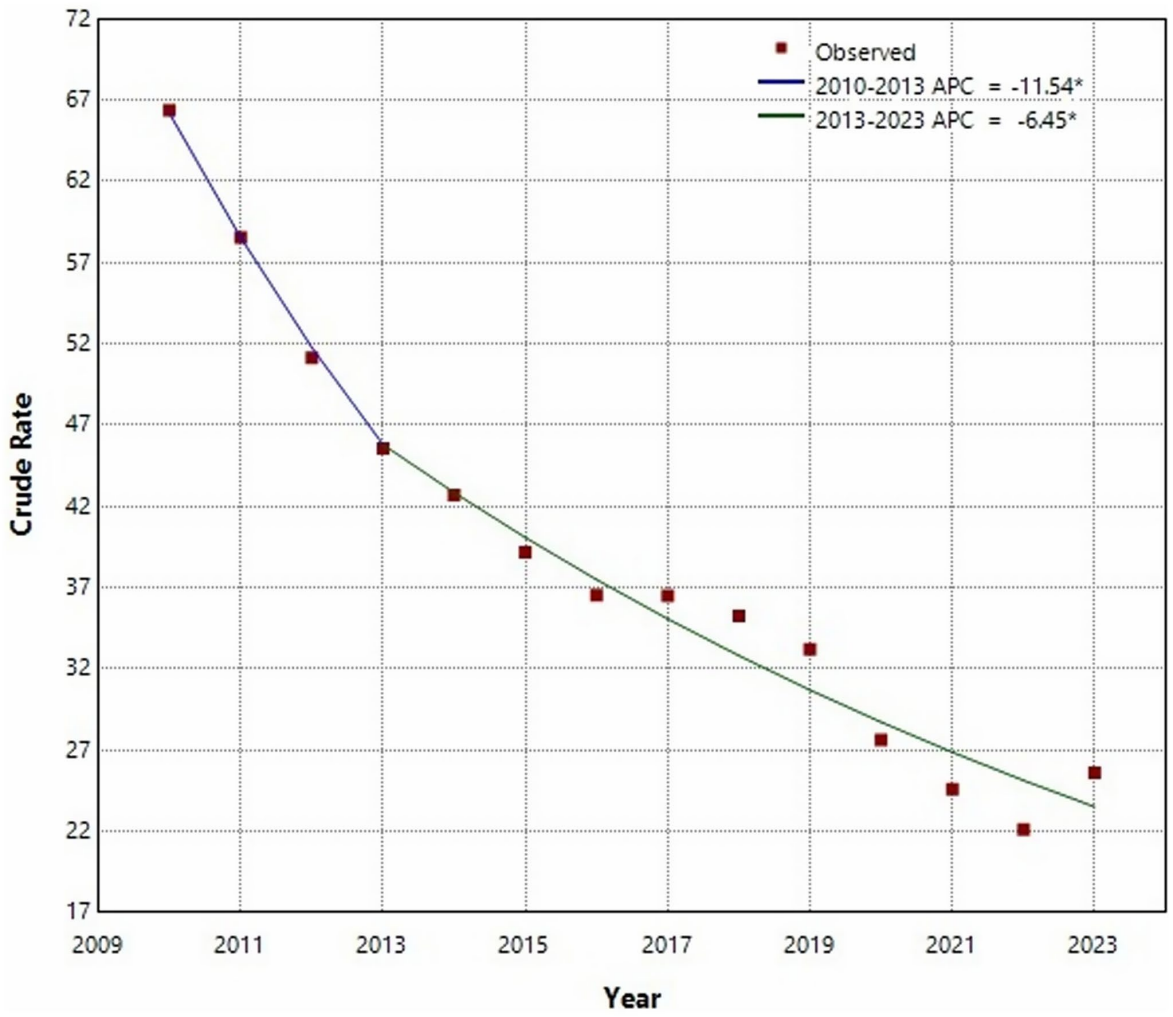
Joinpoint regression analysis of the incidence of pulmonary tuberculosis in Shijiazhuang from 2010 to 2013.

The Joinpoint regression analysis revealed a statistically significant downward trend in PTB incidence from 2010 to 2023 (AAPC = −7.65, 95% CI: −9.47% to −5.79%, *p* < 0.05). A notable inflection point was identified in 2013. From 2010 to 2013, the decline was steep (APC = −11.54, 95% CI: −18.53% to −3.97%, *p* < 0.05), followed by a more moderate but still statistically significant decrease from 2013 to 2023 (APC = −6.45, 95% CI: −8.01% to −4.86%, *p* < 0.05) (Figure 1). In terms of seasonality, PTB cases were reported every month throughout the 14-year period (Supplementary Figure S2). Higher seasonal indices were observed in January and from March to May, indicating peaks in transmission or detection during these months.

**Figure 2:**
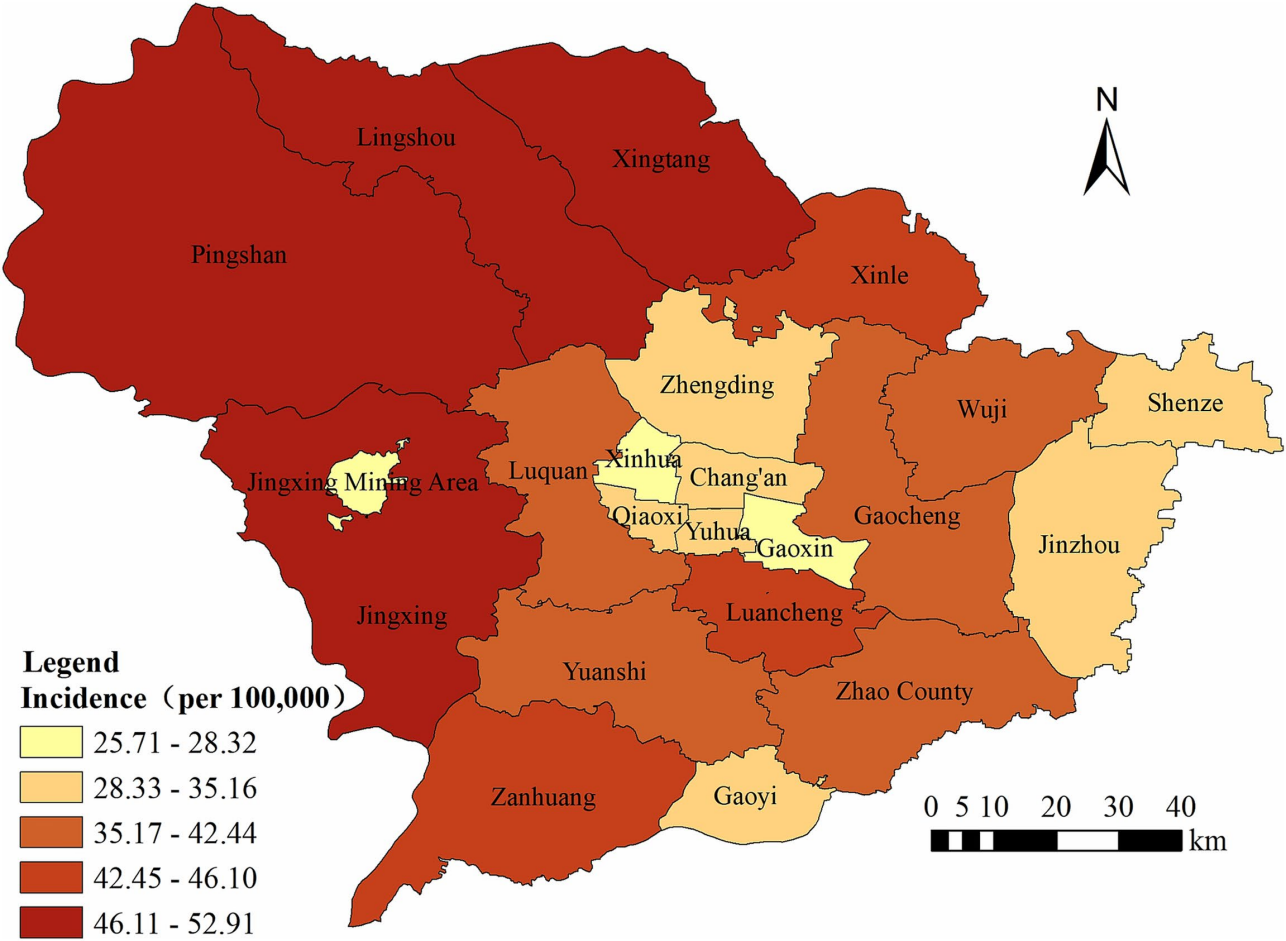
The regional distribution of pulmonary tuberculosis reported in Shijiazhuang from 2010 to 2023.

Finally, we noticed that the annual average PTB incidence varied markedly across the different counties of Shijiazhuang, ranging from 25.71 to 52.91 per 100,000 population. The counties with the highest incidence rates were Xingtang, Lingshou, and Pingshan (Figure 2).

### Spatial autocorrelation analysis

The results of the global spatial autocorrelation analysis of the incidence rate of PTB in Shijiazhuang City from 2010 to 2023 showed that statistically significant positive spatial autocorrelation (*p* < 0.05) was detected in 2011 and from 2017 to 2023, indicating that the incidence rate of PTB in these years presented a non-random clustered spatial distribution among administrative units. The highest degree of clustering occurred in 2020 (Moran’s *I* = 0.680), while the lowest significant clustering was noted in 2011 (Moran’s *I* = 0.300). In contrast, the Moran’s *I* values in other years were non-significant, indicating a more random spatial pattern of disease distribution (Table 2).

**Table 2:** Global spatial autocorrelation analysis of reported incidence of pulmonary tuberculosis in Shijiazhuang from 2010 to 2023.

Year	Moran’s *I* value	*Z* value	*p* value
2010	0.193	1.535	0.125
2011	0.300	2.239	0.025
2012	0.032	0.492	0.623
2013	−0.084	−0.223	0.824
2014	−0.279	−1.410	0.158
2015	−0.058	−0.061	0.951
2016	−0.048	−0.004	0.997
2017	0.348	2.596	0.009
2018	0.676	4.589	<0.001
2019	0.633	4.394	<0.001
2020	0.680	4.656	<0.001
2021	0.499	3.489	<0.001
2022	0.333	2.379	0.017
2023	0.611	4.099	<0.001

**Figure 3:**
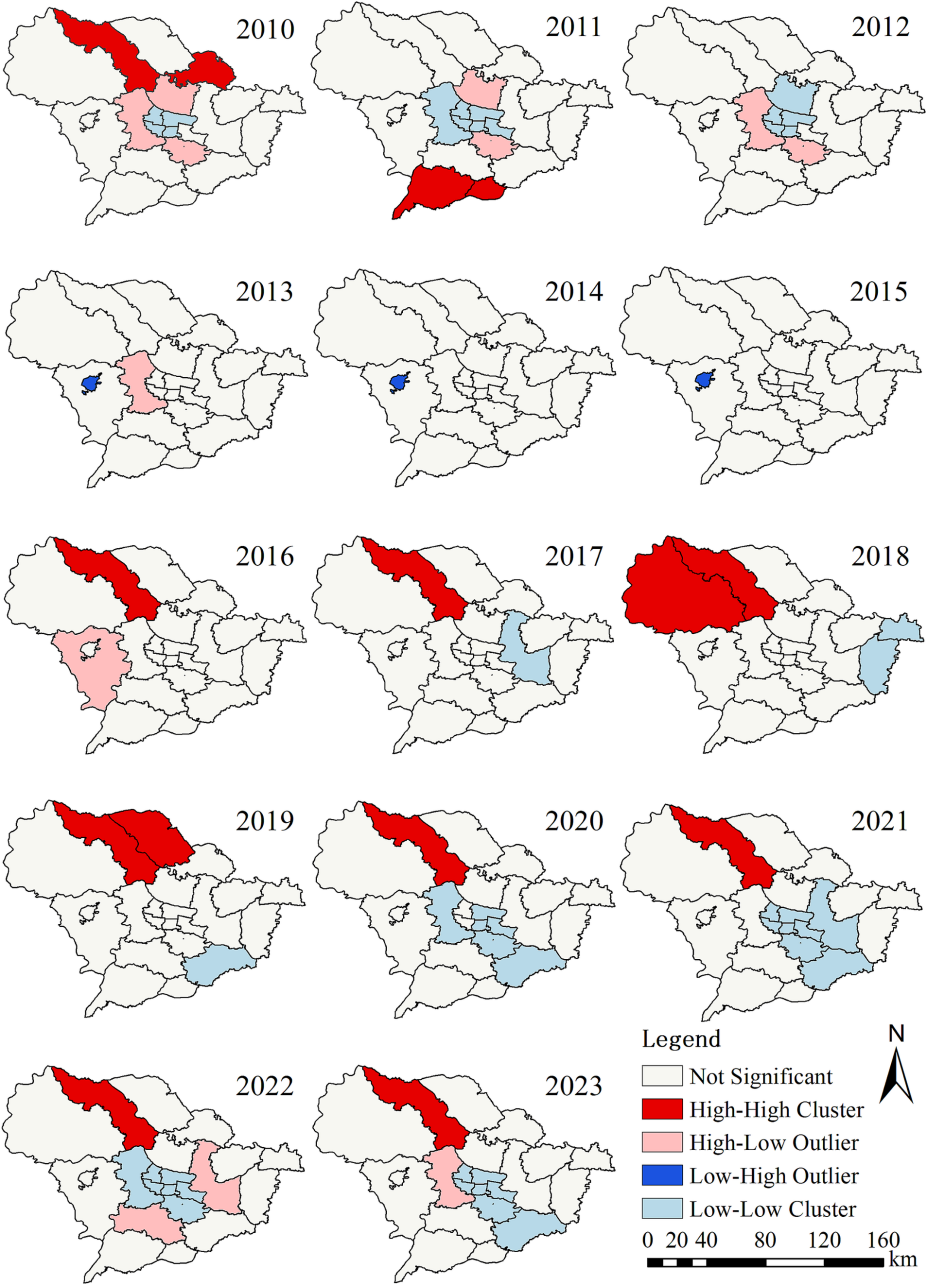
Local spatial autocorrelation analysis of reported incidence of pulmonary tuberculosis in Shijiazhuang from 2010 to 2023.

LISA maps for PTB incidence identified a total of 6 high-high, 12 low-low, 6 high-low, and 1 low-high clusters throughout the study period (Supplementary Table S1; Figure 3). The high-high agglomeration areas are mainly distributed in the northern part of Shijiazhuang, while the low-low agglomeration areas are primarily located in the urban center and surrounding districts. From 2016 to 2023, Lingshou County has consistently been identified as a hotspot for PTB.

### Space–time scan analysis

Spatio-temporal clustering analysis using SaTScan indicated that the notification rates of PTB exhibited spatio-temporal clustering. The results are shown in Table 3. In the first stage, from 2010 to 2017, a total of 12 clusters emerged. The most likely cluster covered two counties, mainly distributed in the southern region, with a clustering period from 2010 to 2012. Eleven secondary clusters were primarily located in the counties surrounding the city center, and the main clustering period was also from 2010 to 2012. In the second stage, from 2018 to 2023, a total of 7 clusters appeared. The most likely cluster involved two counties, with a clustering period from 2018 to 2019 and a total of 1,187 cases reported. The analysis results showed that the PTB risk in these districts and counties was 1.99 times that of non-hotspot districts and counties. Six secondary clusters were distributed in the city center and the northern region, with the main clustering period from 2018 to 2019. Compared with the first stage, the scope of clustering decreased. Notably, a clustering area was identified in 2022.

**Table 3:** Space-time Scan Analysis on reported incidence of pulmonary tuberculosis in Shijiazhuang from 2010 to 2023.

Time period	Cluster type	Number of clustering areas	Cluster districts and Counties	Time frame	Observed cases	Expected cases	Relative risk	LLR	*P* value
2010–2017	Most likely cluster	2	Luancheng District, Zhao County	2010–2012	1,876	1305.77	1.46	114.096	<0.001
	Secondary cluster 1	1	Chang’an District	2010–2012	804	464.64	1.75	103.077	<0.001
	Secondary cluster 2	2	Lingshou County, Pingshan County	2010–2012	1,610	1108.87	1.47	102.726	<0.001
	Secondary cluster 3	3	Gaoyi County, Zanhuang County, Yuanshi County	2010–2012	1,725	1229.63	1.42	91.996	<0.001
	Secondary cluster 4	1	Gaocheng District	2010–2012	1,584	1123.22	1.43	86.687	<0.001
	Secondary cluster 5	2	Shenze County, Wuji County	2010–2011	1,092	722.72	1.53	83.313	<0.001
	Secondary cluster 6	1	Zhengding County	2010–2011	679	449.4	1.52	51.35	<0.001
	Secondary cluster 7	3	Jingxing Mining Area, Luquan District, Jingxing County	2010–2012	1,570	1211.46	1.31	50.276	<0.001
	Secondary cluster 8	1	Jinzhou City	2010–2011	749	515.96	1.46	46.862	<0.001
	Secondary cluster 9	1	Xinle City	2010–2012	955	710.5	1.35	38.757	<0.001
	Secondary cluster 10	1	Jingxing County	2014–2015	380	303.29	1.26	9.053	0.010
	Secondary cluster 11	1	Chang’an District	2013–2013	328	260.55	1.26	8.123	0.016
2018–2023	Most likely cluster	2	Xingtang County, Lingshou County	2018–2020	1,187	617.09	1.99	216.194	<0.001
	Secondary cluster 1	1	Luanquan District	2018–2019	425	268.95	1.59	39.113	<0.001
	Secondary cluster 2	1	Xinle City	2018–2019	393	285.18	1.39	18.543	<0.001
	Secondary cluster 3	1	Yuhua District	2018–2018	227	158.29	1.44	13.262	<0.001
	Secondary cluster 4	1	Chang’an District	2018–2019	563	473.15	1.2	8.272	0.008
	Secondary cluster 5	1	Qiaoxi District	2018–2019	569	480.78	1.19	7.866	0.01
	Secondary cluster 6	2	Lingshou County, Pingshan County	2022–2022	261	205.31	1.28	7.039	0.028

## Discussion

Spatio-temporal analysis is increasingly applied in the study of infectious diseases to detect disease clusters and pinpoint high-risk areas (23–25). Compared to traditional epidemiological methods, it offers distinct advantages, including faster outbreak detection, more efficient resource allocation, and evidence-based policy implementation (26). This study provides a comprehensive epidemiological analysis of PTB in Shijiazhuang from 2010 to 2023, focusing on population characteristics, temporal trends, and spatial distribution patterns. The findings highlight key demographic, temporal, and geographic variations in PTB incidence, providing valuable insights for PTB prevention and control.

The study analyzed 54,855 reported PTB cases over the 14-year period, with a significantly higher prevalence among males than females which similiar with Si Chuan province (27). The observed gender difference in PTB incidence corroborates findings reported in another study from China (28), suggesting that men may be at greater risk due to occupational exposure, lifestyle behaviors such as smoking and alcohol consumption, and biological differences in immune response (29, 30). Notably, 20.76% of cases were aged 65 years and above, indicating that older adults remain a key at-risk population. Age-related immune system decline, comorbidities such as diabetes and chronic lung diseases, and limited healthcare access in some older adult populations may contribute to the increased vulnerability of older adults (31, 32). These findings underscore the importance of targeted TB screening and early intervention programs for older individuals, particularly in settings with an aging population. The occupational distribution analysis shows that farmers dominate the tuberculosis cases, which is consistent with the study of Deng et al. (33). This may be related to the generally limited access to healthcare, poorer living conditions, and greater susceptibility to environmental risk factors that promote the spread of tuberculosis among rural populations (27). These findings suggest that TB screening and health education activities should be prioritized to these population.

Joinpoint regression analysis indicated an overall declining trend in PTB incidence from 2010 to 2023, with a significant turning point in 2013. Between 2010 and 2013, PTB incidence declined rapidly, whereas from 2013 to 2023, the decline was more gradual. The initial rapid decline in PTB incidence may be attributed to strengthened TB control measures, including improved diagnosis, treatment adherence programs, and public health campaigns (34, 35). However, the slower decline after 2013 suggests that while TB control efforts have been effective, further reductions in incidence require additional strategies, such as expanded preventive treatment and improved active case detection. Another important finding was the resurgence of TB incidence in 2023, despite the overall downward trend over the study period. This rebound may be explained by multiple factors. First, the COVID-19 pandemic likely disrupted TB detection and treatment services in preceding years, leading to an accumulation of undiagnosed or untreated cases that surfaced in 2023 (36). Second, increased post-pandemic social mobility and economic recovery may have facilitated TB transmission (37, 38). This resurgence highlights the need for continued vigilance in TB control efforts, particularly in the wake of major public health crises that may indirectly impact TB management.

The seasonal index of PTB incidence from 2010 to 2023 shows that January and the period from March to May are peak months. These seasonal fluctuations are consistent with PTB transmission patterns observed in previous studies (39) and may be linked to factors such as indoor crowding during colder months (40, 41). In addition, this phenomenon is also the case in the Shanghai (11). Understanding these seasonal trends can inform targeted intervention efforts, such as intensified TB screening campaigns during peak months.

The regional distribution of PTB incidence in Shijiazhuang city exhibits significant heterogeneity. The highest burden was observed in Xingtang, Lingshou, and Pingshan counties, all of which are predominantly rural and face structural challenges such as limited healthcare infrastructure, lower socioeconomic development, and poor awareness of TB (42). In addition, Given Shijiazhuang’s proximity to Beijing, tuberculosis recurrence and reactivation remain significant barriers to elimination, partly due to exogenous reinfection risks associated with Beijing family strains, HIV coinfection, imprisonment, and immigration (43). These factors may hinder timely diagnosis and treatment, contributing to sustained transmission in these regions. Such geographic disparities underscore the urgent need for locally adapted interventions, particularly in underserved rural areas. Strategies such as mobile screening services, community-based treatment support, and health education campaigns should be prioritized to improve early detection and treatment adherence.

The global spatial autocorrelation analysis indicates that PTB in Shijiazhuang exhibits a certain degree of spatial clustering. The local spatial autocorrelation analysis further identified that the high-high clustering areas were mainly in Lingshou County, Xingtang County, and Pingshan County, while the low-low clustering was primarily in urban districts such as Chang’an, Qiaoxi, and Xinhua, which is consistent with the regional distribution results. The following facts may explain why these counties and districts have become hotspots. First, these counties and districts are economically underdeveloped areas with a shortage of health resources. In addition, people living in these impoverished counties are mostly farmers, who have low incomes and poor awareness of seeking medical treatment (42, 44). This has led to the continued spread of diseases and the formation of clusters. These findings echo into earlier studies in other regions such as Chongqing (45), indicating that the spatial differences in TB are closely related to the distribution of socio-economic and medical resources. Furthermore, previous studies in Jiangsu have shown that tuberculosis incidence tends to be higher in low-density areas, possibly due to the close association between population density and levels of economic development (46). This suggests that rural areas continue to bear the highest TB burden, emphasizing the need for region-specific TB control strategies, including mobile screening units and community-based treatment support.

The space–time scan analysis results show that over time, the scope and intensity of clustering areas have gradually decreased. The possible reasons for this trend are as follows: First, the TB control efforts in Shijiazhuang have achieved certain results. Second, during the COVID-19 pandemic, strict quarantine measures reduced population aggregation and movement. The widespread use of masks by the public further lowered the transmission risk of MTB, thus forming a positive intervention against the spread of TB. Furthermore, the study demonstrated that PTB transmission exhibits significant spatio-temporal clustering, rather than uniform distribution. These patterns, characterized by peaks in specific time periods and geographic areas, suggest that local outbreaks, population movements, and variations in TB control program efficacy may be driving forces (47). The clustering in earlier years may reflect the combined effects of less advanced tuberculosis control technologies and inadequate resources at that time, as well as the concentrated exposure of high-risk populations (44). These findings emphasize the importance of incorporating spatio-temporal data into TB control plans. Future efforts should focus on strengthening surveillance in known cluster areas, scaling up preventive interventions, and ensuring equitable access to diagnostic and treatment services—particularly in regions identified as recurrent or emerging hotspots.

This study has several strengths. First, it provides a long-term epidemiological assessment of PTB in Shijiazhuang, covering a 14-year period with a large sample size of 54,855 cases. This allows for robust analysis of population characteristics, temporal trends and spatial patterns. Furthermore, by identifying high-risk populations, seasonal trends, and geographic clusters, this study provides actionable insights for TB control and prevention strategies. However, conducting spatio-temporal analysis based on the incidence rate at the county (city, district) level has certain limitations. Analysis at the township level can more precisely identify high-risk areas, which is planned for the next step of this study. Third, the impact of COVID-19 on TB trends remains a complex issue that requires further investigation, particularly in assessing changes in TB diagnosis and treatment adherence during and after the pandemic.

In summary, this study has conducted a comprehensive epidemiological assessment of the epidemiological characteristics, temporal, and spatial distribution of PTB in Shijiazhuang from 2010 to 2023. Although the overall incidence rate of PTB is declining, significant clusters are still evident, such as among susceptible population, in specific time periods, and in certain areas. Our research has identified the spatio-temporal clustering of PTB incidence, with high-incidence clusters mainly concentrated in the northern part of the region. It is recommended that, on the basis of consolidating existing prevention and control achievements, more effective measures be explored to reduce the severity of tuberculosis in key populations and areas.

## Data Availability

The data that support the findings of this study are not openly available due to reasons of sensitivity and are available from the corresponding author on reasonable request.
