# Supplementary figures and images for: Analysis of the epidemiological characteristics of pulmonary tuberculosis in Shijiazhuang, China 2010–2023
Source: Front Public Health. 2025 Jul 3;13:1621695. doi: 10.3389/fpubh.2025.1621695 (PMC12267174; doi:10.3389/fpubh.2025.1621695)

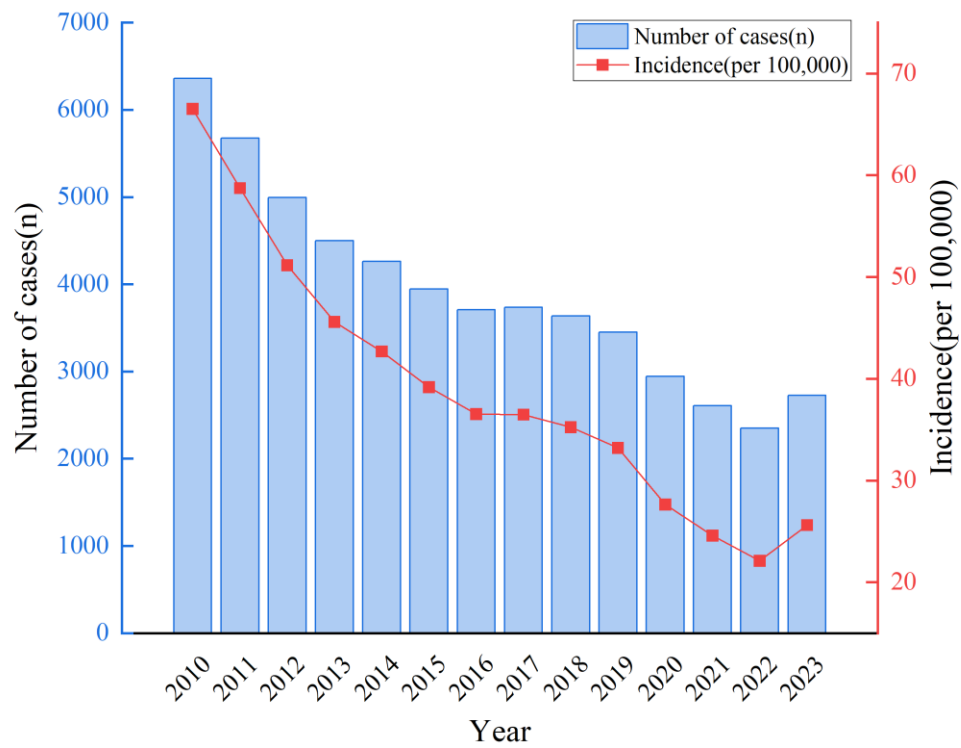

**Supplementary Figure 1** The time distribution of PTB reported in Shijiazhuang from 2010 to 2023

Supplement: Supplementary file 2 [file Data_Sheet_1.PDF]
