# Supplementary material for: Analysis of the epidemiological characteristics of pulmonary tuberculosis in Shijiazhuang, China 2010–2023
Source: Front Public Health. 2025 Jul 3;13:1621695. doi: 10.3389/fpubh.2025.1621695 (PMC12267174; doi:10.3389/fpubh.2025.1621695)
Supplement: Supplementary file 3 [file Data_Sheet_2.PDF]

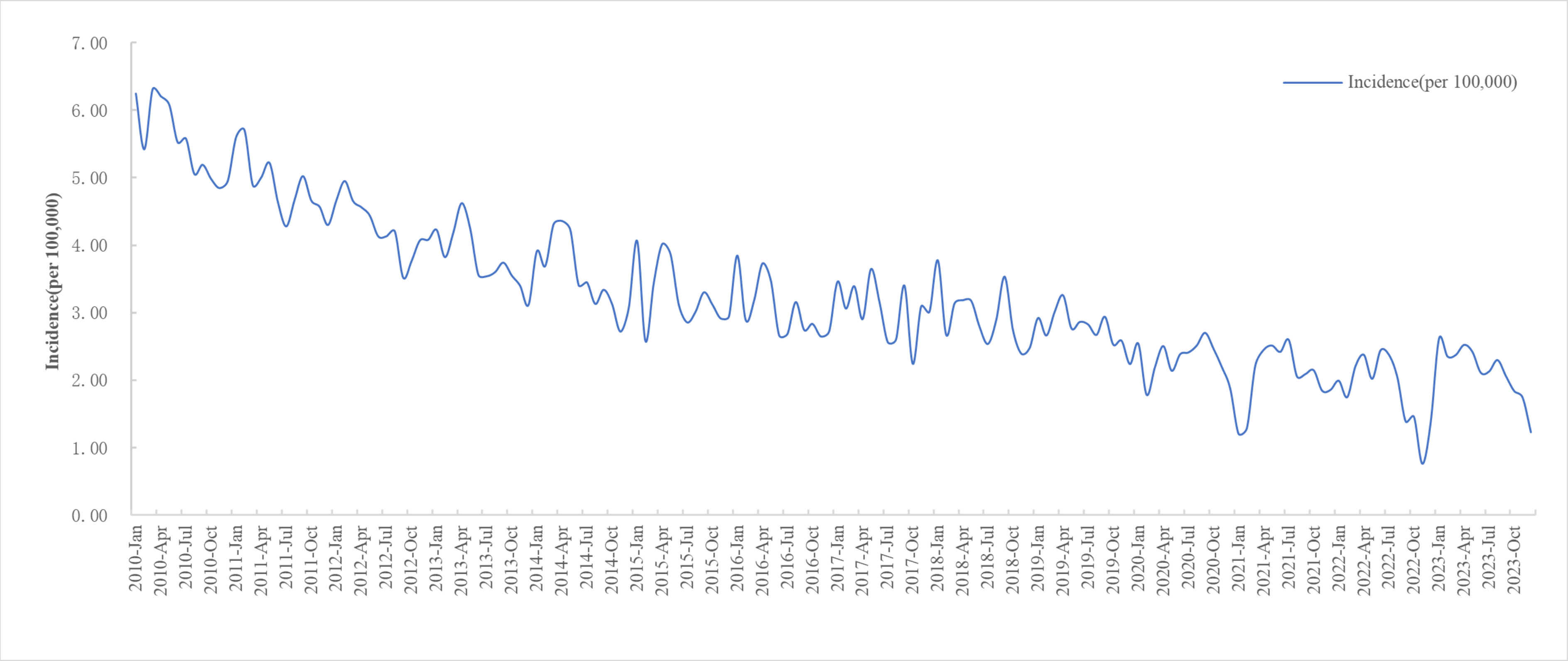

Supplementary Figure 2 Monthly distribution of reported incidence of PTB in Shijiazhuang from 2010 to 2023
